# Expression of macromolecular organic nitrogen degrading enzymes identifies potential mediators of soil organic N availability to an annual grass

**DOI:** 10.1038/s41396-023-01402-3

**Published:** 2023-04-14

**Authors:** Ella T. Sieradzki, Erin E. Nuccio, Jennifer Pett-Ridge, Mary K. Firestone

**Affiliations:** 1 Department of Environmental Science, Policy and Management, University of California, Berkeley, CA USA; 2 Physical and Life Sciences Directorate, Lawrence Livermore National Laboratory, Livermore, CA USA; 3 Life & Environmental Sciences Department, University of California Merced, Merced, CA USA; 4 Present Address: Laboratoire Ampère, École Centrale de Lyon, Lyon, France

**Keywords:** Microbial ecology, Biogeochemistry

## Abstract

Nitrogen (N) is frequently limiting to plant growth, in part because most soil N is present as polymeric organic compounds that are not readily taken up by plants. Microbial depolymerization of these large macromolecular N-substrates gradually releases available inorganic N. While many studies have researched and modeled controls on soil organic matter formation and bulk N mineralization, the ecological—spatial, temporal and phylogenetic—patterns underlying organic N degradation remain unclear. We analyzed 48 time-resolved metatranscriptomes and quantified N-depolymerization gene expression to resolve differential expression by soil habitat and time in specific taxonomic groups and gene-based guilds. We observed much higher expression of extracellular serine-type proteases than other extracellular N-degrading enzymes, with protease expression of predatory bacteria declining with time and other taxonomic patterns driven by the presence (*Gammaproteobacteria*) or absence (*Thermoproteota*) of live roots and root detritus (*Deltaproteobacteria* and Fungi). The primary chitinase *chit1* gene was more highly expressed by eukaryotes near root detritus, suggesting predation of fungi. In some lineages, increased gene expression over time suggests increased competitiveness with rhizosphere age (*Chloroflexi*). Phylotypes from some genera had protease expression patterns that could benefit plant N nutrition, for example, we identified a *Janthinobacterium* phylotype and two *Burkholderiales* that depolymerize organic N near young roots and a *Rhizobacter* with elevated protease levels near mature roots. These taxon-resolved gene expression results provide an ecological read-out of microbial interactions and controls on N dynamics in specific soil microhabitats and could be used to target potential plant N bioaugmentation strategies.

## Introduction

Plants are commonly limited by nitrogen (N) in temperate soils since their access to the largest soil N pools is constrained by the activity of microorganisms responsible for N_2_ fixation and depolymerizing macromolecular organic N compounds [1–3]. Plants thus depend on microbial degradation of complex molecules such as proteins and chitin [4–7] and can potentially increase N availability by releasing exudates that stimulate microbial turnover of organic N pools [8–12]. Depolymerization of high molecular weight detrital organic N is a primary rate-limiting step in soil N mineralization [13, 14] and depends on the activity of extracellular enzymes such as lysozyme, protease, chitinase, nuclease, and urease. The resulting N monomers are taken up by microorganisms and roughly 30% of the amino acid carbon is respired, leading to excretion of amino acids and excess ammonium, which can benefit nearby plants [15–18]. While the bulk-scale activity of extracellular N-degrading enzymes in soil is well-studied, N mineralization is underpinned by an expansive suite of enzymes whose spatial, temporal, and phylogenetic gene expression dynamics are unknown. Additionally, soil microorganisms with the genomic capacity for organic N degradation are phylogenetically diverse [19], and it is unclear how niche partitioning occurs [20].

Many soil organisms, living in diverse physicochemical niches, are involved in mineralization of organic N. However, most extracellular enzyme assays treat the soil as a “homogenous medium” [13] and average across microhabitats. Until recently, pure culture studies or targeted marker gene surveys [7, 21] were needed to link the type and activity of specific N-depolymerization enzymes to specific prokaryotic lineages, although recent studies [19] have illustrated how peptidases are patterned across different proteolytic super-families with comparative genomics, highlighting the role phylogeny might play in the activity of these enzymes in the environment. Measurements of community mRNA transcripts can reflect enzyme production rates better than DNA functional gene concentrations [22]. Transcripts can be resolved by taxonomy, thus illuminating the ecological and evolutionary factors that regulate activity of specific lineages. This extends to include microbiome interactions, such as activity of bacterial predators (e.g., *Myxococcales*, *Bdellovibrionales*, *Cytophagia*) or fungivores, grazing of rhizosphere protozoa on bacteria that leads to enhanced plant root N availability [12], or the breakdown of chitin-rich microarthropod and fungal biomass. Gene transcript patterns could also help to identify the specific lineages involved in plant-triggered priming or protease inhibitors [7, 23]. Finally, measurements of specialized peptidase functions and taxonomic optimization could aid in the identification of plant N bioaugmentation microbes, based on their spatiotemporal patterns of protease gene expression.

Patterns and controls of soil N depolymerization and mineralization may be highly dependent on soil habitat (e.g., rhizosphere, detritusphere, bulk soil), since availability of organic-N substrates and the prevalence of fungal, faunal and bacterial degraders varies in both time and space [23, 24]. In the rhizosphere, organic N is available as amino acids, niacin, choline (derived from plant exudates [11]), lignoproteins and aromatics (from sloughed off cells), nucleic acids and microbial cell wall amino sugar polymers (N-acetylglucosamine, N-acetylmuramic acid) derived from the bloom of cells that develops as roots grow [20]. Prior work has found much higher N-compound enzyme activities in rhizosphere soils compared to bulk soil [25] and shown that root exudates can increase degradation of soil organic matter by up to 380% [26]. Meanwhile, the detritusphere (soil-root litter interface) tends to have highly heterogeneous rates of proteolytic activity compared to bulk soil [27]. Bulk protease enzyme activity has been shown to be enhanced by both leaf litter addition [28] and root exudates [29].

Soil N pools and by proxy, N mineralization activity, change with time [30] in part due to the succession of both exudate quality and microbial communities [11, 31]. At the bulk soil level, activity of extracellular proteases is thought to drive soil N cycling [13], but what controls this activity is not always clear [32]. Ecological patterns with time and soil habitat are likely confounded because most studies are conducted on whole soils, which contain a mixture of rhizosphere, detritusphere and bulk regions. In grassland soils, the rhizosphere is a particularly critical hotspot for microbial activity due to rapid recycling of root exudation and debris and functions as a quasi-digestive system decomposing molecules inaccessible to plants. However, root exudation, which serves as a carbon source for rhizosphere bacteria, declines near older root sections [33]. Therefore, it has been hypothesized that bacteria in a mature rhizosphere environment may be forced to target less labile, higher C:N sources of organic N such as plant litter [34]. Indeed, it has been shown that the mature *Avena* sp. rhizosphere has higher rates of gross nitrogen mineralization and ammonia consumption compared to the young rhizosphere [28].

The vast majority of studies of soil N depolymerization have been conducted with enzyme activity assays, with a lesser number using isotope tracing approaches [23, 35], gene abundance measured either via qPCR [21, 36], GeoChip [37] or metagenomics [21, 38] and for chitin, with activity-based probes for profiling pure culture active chitinolytic enzymes [39] and via gene expression in model consortia [40]. While common, specific enzyme activities (such as protease and urease activity) cannot identify the microbial taxa involved and several concerns have been raised about the variable application of enzyme assays [41] and their reliability [7, 35, 42]. Bulk assays also cannot resolve links between specific microbial community members and the factors that shape their enzyme regulation. Some have suggested that a genetic approach to determine protease gene expression would be ideal [19, 41] and Ouyang et al. [36] showed that the abundance of functional genes was significantly correlated with their corresponding enzyme activity. However, to our knowledge, no previous studies have used metatranscriptome-based gene expression to link the ecology of N mineralization enzyme activity to microbial taxonomy and soil habitats.

To determine spatial and temporal patterns and identify the soil microorganisms primarily responsible for decomposition of complex N-containing molecules, we assessed the expression of genes that code for enzymes degrading macromolecular organic N near actively growing and decomposing roots of a common annual grass *Avena fatua* (wild oat grass). As compared to domestic oat, the wild oat in California grasslands is an ideal source for putative beneficial microorganisms since it does not experience fertilization and therefore needs to acquire N from soil organic matter via its microbiome. We analyzed 48 metatranscriptomes collected over three weeks of active root growth, using a well-developed plant mesocosm approach [31, 34, 43, 44] with a fully-characterized Northern California annual grassland soil. A separate subset of these metatranscriptomic data was previously used to analyze expression of genes coding for degradation of carbohydrates, in an effort to determine the effect of roots and root litter on carbon cycling [28]. While this previous study provides important context, our analysis here explores how microbial spatial and temporal dynamics control taxon-specific expression of enzymes that are key to macromolecular N-availability to plants. We also sought to differentiate which proteases and chitinases were involved in microbiome interactions.

## Materials and methods

### Experimental design, sample collection, and sequencing

The experimental design, sample collection and sequence data processing are described in detail in ref. [44]. Briefly, common wild oat *Avena fatua* was grown for six weeks in rhizobox microcosms containing soil from the Hopland Research and Extension Center (HREC) in northern California, a Bearwallow–Hellman loam (pH 5.6, 2% total C) packed at field bulk density. Roots were then allowed to grow into a sidecar soil region with a transparent wall, where the root growth timeline was marked at 3 days, 6 days, 12 days and 22 days. In half of the sidecars, the soil was amended with dried *A. fatua* root detritus (C:N = 13.4) chopped to 1 mm. Bulk soil bags, inaccessible to roots, were placed in each sidecar; a bulk soil treatment amended with root detritus was also included. At each timepoint, three replicate microcosms were destructively harvested for paired rhizosphere and bulk soil, both detritus amended and unamended, yielding a total of 48 samples representing four treatments at four timepoints: rhizosphere, rhizosphere + root litter, bulk, and bulk + root litter (Fig. S1).

Harvested soil (1 g) was placed immediately in Lifeguard Soil Preservation Reagent (MoBio) and processed according to the company protocol. Roots and supernatant were removed and the soil was stored in −80 °C. DNA and RNA were co-extracted using a phenol-chloroform procedure [45, 46] and separated with an AllPrep kit (Qiagen). RNA was DNase treated (TURBO DNase, Thermo-Fisher Scientific), depleted in ribosomal RNA (Ribo-Zero rRNA Removal Kit, Epicenter) and reverse transcribed into cDNA. Metatranscriptomes were sequenced for 48 samples on an HiSeq 2000 2 × 150 (Illumina TruSeq SBS v3) at the Joint Genome Institute (JGI), in Walnut Creek CA.

### Expression of macromolecular N degrading genes identified in de novo assembled metatranscriptomes

Raw reads were quality-trimmed (Q20) and rRNA and tRNA reads were removed. Library size, evenness, richness and Shannon diversity were comparable between experimental groups with a mean library size of 43 M paired end reads after filtering. In contrast to the single approach used previously [28], we also de novo assembled quality-controlled metatranscriptomic reads into contigs within each sample. Contigs smaller than 200 bp were discarded and the remaining contigs from all samples were clustered at 99% identity with cd-hit-est keeping the longest sequence as the cluster representative [47]. Open reading frames (ORFs) were predicted using Prodigal [48]. Extracellular protease ORFs were identified by reciprocal BLAST to extracellular peptidases from the MEROPS database [19, 49]. We acknowledge that this method may generate a conservative estimate of the number of proteases detected. ORFs were assigned a peptidase group (serine-, metallo-, cysteine-peptidase and others) by their best reciprocal BLAST hit. Taxonomy was determined by best BLAST hit to the NCBI non-redundant database (NR, access date July 29th 2019). Chitinase ORFs were identified using six chitinase hidden Markov models (HMMs) from the Kyoto Encyclopedia of Genes and Genomes (KEGG): K01183, K03791, K13381, K17523, K17524, and K17525. Only the first three were detected in our dataset. Lysozyme ORFs were identified using the HMM for KEGG orthology K07273, extracellular nuclease with K01150 (codes for Dns gene, undetected) and K07004, and urease subunit A, B and C with K01428, K01429, and K01430 respectively. Reads were then mapped back to ORFs requiring minimum identity 95% and 75% breadth using bbmap [50]. Read counts were normalized using DESeq2 [51]. Heat maps of normalized counts were generated in R using the heatmap2 function in gplots [52]. Normalized transcript counts per gene per time point were compared between groups (rhizosphere, root litter and root litter-amended rhizosphere) using ANOVA and Tukey post-hoc test. Boxplots were generated in ggplot2 [53].

### Expression of extracellular protease genes from a curated collection of Hopland-soil genomes

The quality-controlled reads were mapped using BBsplit [50] requiring 80% identity against a dereplicated reference database of 282 HREC soil genomes including isolates [11], single amplified genomes (SAGs) [44], metagenomic assembled genomes (MAGs) (NCBI PRJNA517182) and stable isotope probing MAGs (SIP-MAGs) [54]. On average, 12.3% (range 6.2–31.9%) of the reads per library mapped unambiguously to genomes from the reference database. This additional approach was taken to investigate gene expression within the context of a genome and to search for overlap in guild membership between extracellular protease defined guilds and previously defined CAZy guilds [44]. Three MAGs that were classified as unknown or domain bacteria in previous studies were assigned taxonomy using GTDB-Tk version 1.3.0 [55, 56]. Verification of the taxonomic assignment of MAG Burkholderiales_62_29 was done using GToTree [57] with complete reference genomes of *Gammaproteobacteria* (formerly *Betaproteobacteria*) from RefSeq (Feb 28, 2020).

ORFs were predicted in all genomes using prodigal [48] and annotated using KEGG [58] and ggKbase (http://ggkbase.berkeley.edu). Extracellular proteases, which do not have hidden Markov models (HMMs) capable of separating them from intracellular proteases, were identified by gene nucleotide identity of at least 90% and coverage of at least 60% to de novo assembled ORFs of extracellular protease from the metatranscriptomes. Gene counts were identified using Rsubread featureCounts [59].

Differential expression of chitinases in the genome collection was already performed in a previous publication on expression of carbohydrate active enzymes (CAZy) and was therefore not repeated here [44].

### Statistical analyses

All features and their abundance (represented by metatranscriptomic read counts normalized to sequencing depth) were analyzed with DESeq2 [51] requiring *p* < 0.05 (adjusted for multiple comparisons). Ordination and visualization were conducted in R using ggplot2 [53] and vegan [60]. PERMANOVA (vegan function adonis) was used to detect significant treatment factors affecting expression of nitrogen depolymerization genes (protease expression by location (proximity to live roots) * treatment * time). We define “guilds” as groups of taxa with similar gene upregulation patterns of extracellular proteases in both time and soil habitat. Guilds were assigned by hierarchical clustering based on differential expression of extracellular proteases compared to unamended bulk soil, generating four functional guilds: “Rhizosphere”, “Detritusphere”, “Aging root” and “Low response”. Effects of the four experimental treatments (bulk, rhizosphere, root litter and root litter-amended rhizosphere) on protease gene expression were assessed by ANOVA (*p* adjusted for multiple comparisons), *N* = 3 per time point.

## Results

### Expression of enzymes targeting macromolecular N

Extracellular enzymes that depolymerize macromolecular N in soil mainly target proteins, cell wall components and nucleic acids [3]. We identified multiple genes for these functions in our analysis, including: extracellular nuclease (*xds*), urease (*ureABC*), lysozyme targeting peptidoglycan (*lys*), chitinase (*chit1*) that targets fungal cell walls and insect exoskeletons and extracellular proteases. Expression of extracellular proteases was an order of magnitude higher than the other extracellular N degrading enzymes (Fig. S2) and like chitinases, was affected by the presence of root litter and roots. In contrast, extracellular nuclease, urease and lysozyme were not influenced by either living or decaying roots, thus, we chose to focus a more detailed analysis on extracellular proteases and chitinases.

**Fig. 1:**
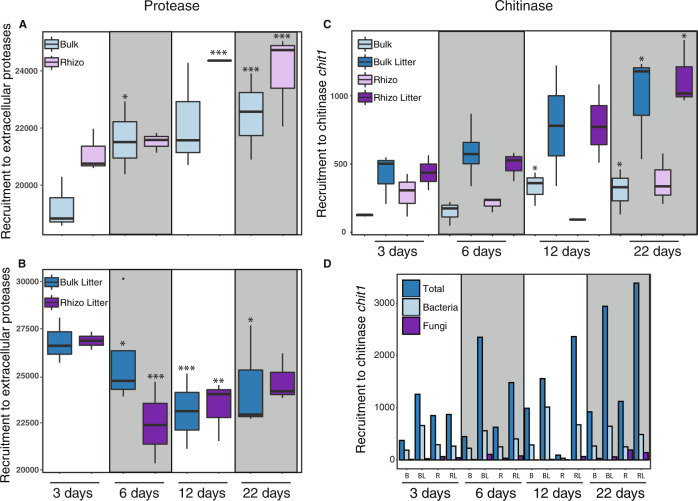
Aggregated expression of extracellular proteases in bulk and rhizosphere soils from common wild oat, *Avena fatua,* microcosms. The plants were grown for 22 days without root litter amendment (**A**), or with litter amendment (**B**); expression of chitinase gene *chit1* (**C**) and mean counts of chitinases at the domain level (**D**) for unamended bulk soil (B), litter-amended bulk soil (BL), unamended rhizosphere (R) and litter-amended rhizosphere (RL). Boxplots represent 75% of the data, whiskers denote 90% of the data and dots (e.g., **B** bulk litter 6 days) represent outliers. Note that the scale of the *Y* axis varies between panels. Statistical significance of linear regression compared to 3 days within the same location and treatment group: *p < 0.05, **p < 0.01, ***p < 0.001.

Transcript abundance normalized to sequencing depth and gene length for 4,948 extracellular protease genes from bacteria (4846) and fungi (102) was significantly affected by time (3-way PERMANOVA, *F* = 2.8, *p* = 0.038), litter amendment (*F* = 118.9, *p* < 0.001) as well as interactions between time:treatment (*F* = 26.9, *p* < 0.001), time:location (*F* = 7.7, *p* < 0.001) and location:amendment (*F* = 15.5, *p* < 0.001). Expression in unamended soil (no litter) generally increased slightly over time (Fig. 1A), whereas root litter-amended soil had initially high expression that then decreased and leveled off over time (Fig. 1B).

The 73 distinct chitinase transcripts were expressed at a substantially lower level than the extracellular proteases (Fig. S2). The expression of the *chit1* gene increased over time in litter-amended soils and was higher than in the unamended treatments (3-way PERMANOVA, *p* < 0.001) (Fig. 1C). Expression of fungal chitinases was lower than bacterial chitinases and both were generally lower than eukaryotic chitinases (Fig. 1D). Transcripts for chitinase KEGG orthologs *CHI3L1_2*, *CHI3L1_4* and *chiA* were not detected at all and the putative chitinase K03791 had low expression and no significant effects with time or treatment (data not shown). Chitinase CHID1 was detected but was most closely related to plants and therefore disregarded.

### Structural groups of extracellular proteases

**Fig. 2:**
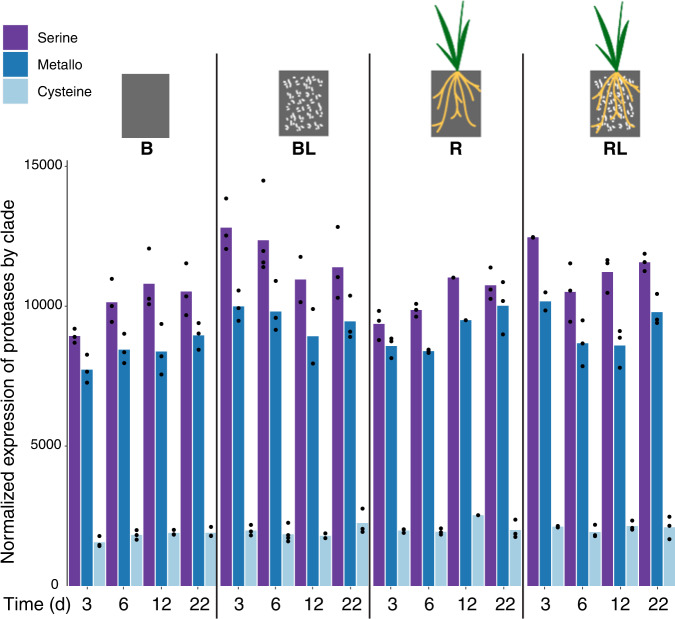
Aggregated normalized expression of extracellular protease structural groups over time in wild oat *Avena fatua* microcosms with bulk, rhizosphere and root litter-amended soils. Normalized expression per replicate is plotted as dots on top of the bars. Experimental groups shown per time point are (left to right): unamended bulk soil (B), litter-amended bulk soil (BL), unamended rhizosphere (R) and litter-amended rhizosphere (RL). Extracellular protease groups are (left to right): serine-, metallo- and cysteine-protease.

The main groups of extracellular proteases found in our assembled metatranscriptomes were serine-, metallo- and cysteine-proteases (2679, 1949, and 496, respectively out of 5295 variants clustered at 99% nucleotide identity). Aggregated expression patterns of all variants in each group reflected the same order (serine > metallo > cysteine). Expression of serine- was consistently higher than metallo-protease across all treatments (ANOVA F = 2392, *p* = 0) (Fig. 2, Table S1). Expression of serine-proteases was also significantly greater in the presence of root litter compared to no litter (diff = 2337, *p* < 0.001), but root litter amendment did not affect expression of metallo- or cysteine-proteases and there was no significant effect of time or location on these structural groups (Table S1). Furthermore, we investigated the relative contribution by various bacterial and archaeal phyla to the expression of the main three structural groups of proteases (Supplementary Tables S5, S6) and found that *Actinobacteria* and *Thermoproteota* (formerly *Thaumarchaeota)* contributed more to expression of metalloproteases than to serineproteases (Fig. S3A). Expression of serine- and cysteine-proteases was dominated by *Proteobacteria* (generally >50%) (Fig. S3B). *Acidobacteria* contributed similarly to expression of metallo- and serine-proteases and at the last time point we noted an increase in *Acidobacteria* cysteinprotease expression. Finally, *Planctomycetes* expressed mostly serineproteases (Fig. S3).

### Taxonomy of extracellular proteases

De novo assembled ORFs of extracellular proteases were taxonomically assigned by BLASTP best hit against the NCBI non-redundant database (NR). Since extracellular proteases are not marker genes and potentially lack high taxonomic resolution, we considered taxonomic assignments only at the order level or higher.

**Fig. 3:**
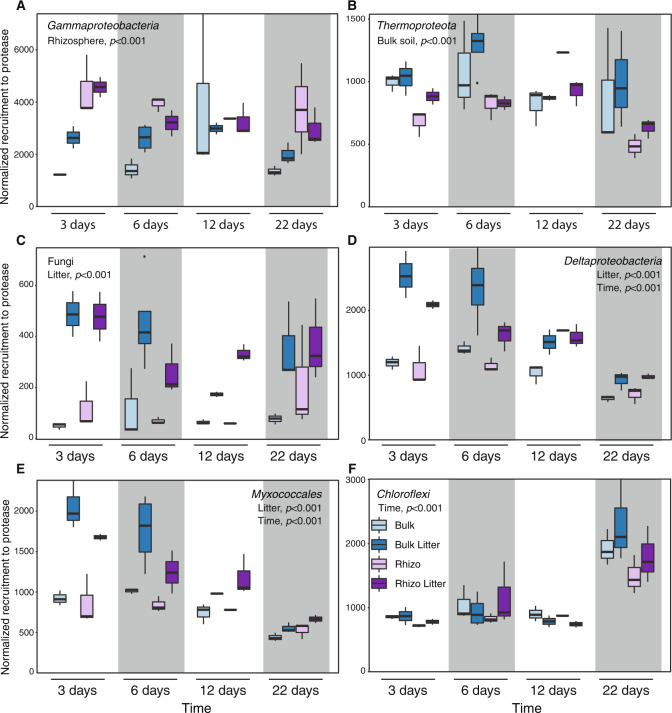
Expression of extracellular proteases in wild oat *Avena fatua* microcosm soils over 22 days of plant growth, for select microbial taxonomic groups. Proteases were divided into taxonomic groups: **A** *Gammaproteobacteria* (formerly *Betaproteobacteria*) (*n* = 547); **B** *Thermoproteota* (formerly *Thaumarchaeota*) (*n* = 112); **C** Fungi (*n* = 99); **D** *Detaproteobacteria* (*n* = 228); **E** *Myxococcales* (*n* = 147); and proteases of (**F**) *Chloroflexi* (*n* = 153). The legend in **F** applies to all panels. Note that as expression levels varied over taxonomic groups and to emphasize patterns, the *y* axes do not have the same scale. Boxplots represent 75% of the data, whiskers denote 90% of the data and dots represent outliers.

In the rhizosphere, *Gammaproteobacteria* (formerly *Betaproteobacteria*) proteases were significantly upregulated (ANOVA, *p* < 0.05) at all time points except 12 days (Fig. 3A). Of 547 variants of proteases from this class, 442 were assigned as *Burkholderiales*. In contrast, at most timepoints, proteases of *Cyanobacteria* and *Thermoproteota* (formerly *Thaumarchaeota*) were significantly upregulated in bulk soil compared to the rhizosphere with the exception of 12 days (Fig. 3B; Table S2). In multiple other taxonomic groups, proteases were significantly upregulated only in the presence of litter: Fungi (Fig. 3C), class *Deltaproteobacteria* (Fig. 3D), as well as highly represented phyla *Bacterioidetes* and *Verrucomicrobia* (Fig. S2; Table S2) and classes *Chitinophagia* and *Gammaproteobacteria* (Table S3). Protease expression declined with time for the predatory bacteria *Myxococcales* (Fig. 3E), *Bdellovibrionales* and *Cytophagia*, while clades such as *Chloroflexi* (Fig. 3F) and *Actinobacteria* had increased protease expression at the final sampling point. *Actinobacteria* and *Acidobacteria* had a high number of variants and high expression of proteases, but we did not detect a significant effect of either time or soil habitat/ amendment. Normalized protease expression data are summarized by phylum (Fig. S4), class (Fig. S5) and order (Fig. S6) and ANOVA *F* and *p* values are in Tables S2, S3 and S4, respectively.

### Functional guilds

**Fig. 4:**
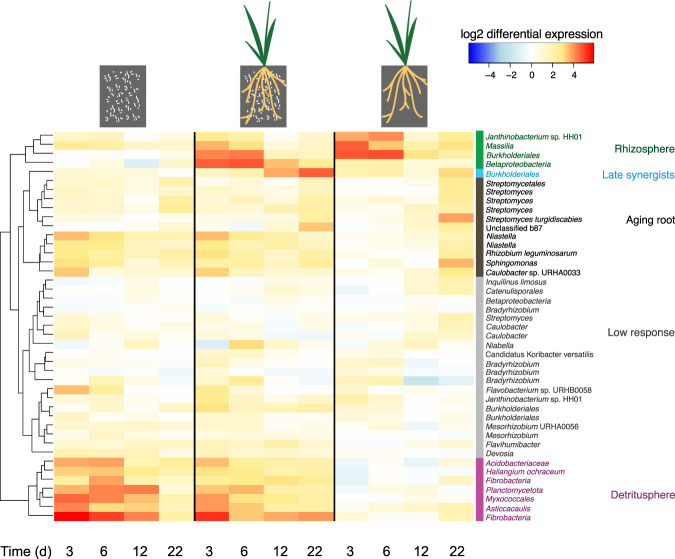
Microbial functional guilds defined by hierarchical clustering of extracellular protease differential gene expression during a 22-day *Avena fatua* microcosm experiment. Each row represents the mean differential expression of extracellular protease genes mapped to a reference genome. Treatments are (left to right): litter-amended bulk soil, litter-amended rhizosphere and unamended rhizosphere. Time points are indicated at the bottom in days. We note that a genome may contain more than one protease gene and that reads were mapped at 80% identity, therefore, each genome also represents closely related taxa. Differential expression values per gene that were not statistically significant were converted to zero (0) before averaging.

To define functional guilds with a population-centric analysis, we mapped transcriptome reads to a collection of 282 genomes and metagenome-assembled genomes (MAGs) generated from the same soil and site [44]. Read counts were used to determine differential expression compared to unamended bulk soil. Each genome contained multiple genes coding for extracellular proteases. Hierarchical clustering of the mean differential expression of extracellular protease genes within each genome was used to define four functional guilds: “rhizosphere”, “detritusphere”, “aging root” and “low response” (Fig. 4). These guilds include bacteria that upregulate expression of extracellular proteases in the presence of live roots (rhizosphere), dead roots (detritusphere), live roots that are beginning to senesce (aging root) or at low but significant levels without a specific pattern (low response) [44]. Similarly, hierarchical clustering based on differential expression of the most highly upregulated de novo assembled ORFs compared to unamended bulk soil revealed clear rhizosphere and detritusphere guilds (Fig. S7). There was limited overlap between guilds identified here and guilds previously identified based on expression of CAZy genes [44], but the degree of overlap varied by guild (Fig. S8).

Within the aging root guild, we noticed one member, representing an aggregated population transcriptome, that increased differential expression of extracellular proteases at the last time point in the presence of both living roots and root litter more than would have been predicted by the sum of the treatments alone. As this pattern differed from the rest of the guild, we identified this member as a sub-guild labeled “late synergist” due to its expression pattern. This MAG, *Burkholderiales*_62_29, was identified by two independent phylogenomic analyses as *Rhizobacter*. *Burkholderiales*_62_29 has 16 different extracellular protease genes, all of which had similar significant upregulation patterns. While the sub-guild here contained only a single *Rhizobacter* MAG, the implication of mapping transcriptomic reads at 80% identity is that each MAG represents a taxonomic “cloud” of at least genus-level diversity. A 16S rRNA gene survey of the same samples revealed 10 operational taxonomic units (OTUs) of order *Burkholderiales,* which, like *Rhizobacter*, are not assigned a family [44]. Additionally, this sub-guild may include more taxa for which we have no MAGs and, therefore, could not be included in this analysis.

We identified potential candidates for bioaugmentation based on increased expression of extracellular proteases in the rhizosphere as (1) a *Janthinobacterium* strain that contains 21 proteases, (2) a *Massilia* with 48 extracellular proteases and (3) a taxon of *Burkholderiales* with 26 extracellular proteases and (4) a taxon of *Gammaproteobacteria* (formerly *Betaproteobacteria*) with 5 extracellular proteases, all of whose expression is upregulated early in rhizosphere development but decreased over time, in contrast to that of *Rhizobacter* proteases (Fig. 4).

## Discussion

Our current understanding of macromolecular nitrogen depolymerization by microorganisms in soil is based almost entirely on measurement of bulk mineralization rates, soil enzyme assays and gene abundance data from genomic surveys. Results from soil enzyme assays using artificial substrates and colorimetric and fluorometric methods can be variable depending on how the methods are applied [41] and are typically conducted in conditions very different from the environment experienced by soil microorganisms. Linking bulk process rates and genetic potential is also challenging, since rates cannot be resolved by taxonomy and gene presence represents only a blueprint which may or may not be acted upon. For example, of the many organic N-degrading enzymes we detected, only protease and chitinase were differentially expressed between soil habitats. In addition, overall microbial community composition changes at a slower rate compared to functional gene expression [44].

Structural groups of extracellular proteases can be soil-specific and pH dependent [61]. Based on genomic potentials [19], we would have hypothesized that expression of structural groups of proteases would be dominated by metalloproteases [19]. However, the expression of serineproteases proved to be significantly higher across our experimental treatments. We found that the main phyla expressing serineproteases and metalloproteases were *Actinobacteria* and *Proteobacteria*, respectively. While there was no significant difference in the taxonomic distribution of expression of each structural group between habitats within each time point, we observed changes over time. For example, the expression of cysteineproteases by *Acidobacteria* increased over time, as did expression of metalloproteases by *Actinobacteria*. Therefore, to resolve the microbial ecology that underlies N mineralization and plant N availability, we suggest that a taxon-resolved transcriptomic approach is crucial.

Expression of extracellular proteases was an order of magnitude higher than the other extracellular N degrading enzymes and like chitinases, was affected by the presence of both root litter and growing roots. Proteins represent the dominant input of organic N into soils and comprise the largest stock of N in soil organic matter [62]. Thus, the breakdown of proteins to amino acids by extracellular proteases is central to plant N-availability and is proposed to be the rate limiting step of N-cycling in soil [62, 63]. Soil proteases have been identified as “the single enzyme most responsible for supplying bioavailable N” [13]. While the role of chitinase in N mobilization cannot be differentiated from its role in C mobilization, its activity has been shown to be positively correlated to C mineralization rates, implying that this activity supports growth [64]. Moreover, activity of protease was an order of magnitude higher than that of chitinase in temperate forests [64], aligning with our gene expression levels. The preference of proteins (C:N = 3.6 [65]) to chitin (5.5 < C:N < 7.5 based on degree of deacetylation [66]) aligns with the microbial N mining hypothesis, which posits that if the community is N limited (particularly in the presence of C sources such as plant litter or exudates), production of enzymes that target polymers with a lower C:N ratio should be increased [67, 68].

We resolved several types of microbiome interactions based on degradation of proteins and chitin. Spatiotemporal niche preferences by protease expression, such as increased expression by *Thermoproteota* (formerly *Thaumarchaeota*) in bulk soil, by *Gammaproteobacteria* (formerly *Betaproteobacteria*) in the rhizosphere and by *Deltaproteobacteria* and fungi in the detritusphere imply effects of both bottom-up controls (higher resource requirements) and ability to compete over substrate. Increased expression of proteases by predatory bacteria of the order *Myxococcales* in the detritusphere may indicate predation of bacteria that use litter as a C and/or N source, although predatory bacteria may also function as opportunistic saprotrophs [69]. Chitinase gene expression, while two orders of magnitude lower than that of proteases, increased over time and was significantly higher in root litter-amended samples. This effect could imply that the litter attracted saprotrophic fungi and possibly arthropods, the cell walls of which are rich in chitin [70]. Indeed, we found that the expression of fungal proteases was significantly higher in the presence of root litter. As most of the chitinases we observed were linked to eukaryotic enzymes, it is possible that in the detritusphere, chitin-rich fungal hyphae were being preyed upon by soil fauna.

In the absence of root litter, expression of extracellular proteases was higher in the rhizosphere compared to bulk soil, potentially because labile carbon inputs from growing root exudates created a higher demand for N [64]. This pattern may also reflect microbial competition for inorganic nitrogen with the plant roots, which forces microbes to turn to organic nitrogen sources [71]. Similarly, DeAngelis et al. showed that protease specific activity was significantly higher in the young rhizosphere compared to bulk soil, but activity (based on enzyme assays) was not significantly different based on root age [43]. In litter-amended soil, expression of extracellular proteases was highest (regardless of root presence) at 3 days, suggesting that litter-added carbon overwhelms the effect of root exudates at this early point in time. At 12 and 22 days there was no difference between litter-amended and unamended soil, indicating that the effect of the root litter had waned, likely due to substrate degradation. In support, expression of carbohydrate active enzymes after litter addition generally decreased over time, implying reduced substrate availability [44].

We identified four main functional guilds based on spatiotemporal expression of protease genes in a curated collection of microbial genomes from the same soil. Hierarchical clustering by extracellular protease expression revealed taxa specializing in the rhizosphere, detritusphere and aging root environments. The rhizosphere guild, as expected, comprised mainly of *Gammaproteobacteria* (formerly *Betaproteobacteria*), which are common members of the *Avena* rhizosphere microbiome [31]. The detritusphere guild was much more diverse, as was the aging root guild (bacteria that increased expression of proteases at the last time point in the rhizosphere). These guilds were clustered closely, likely because the live roots were beginning to decompose towards the end of the experiment and resembling root litter. Indeed, the last time point of our experiment differed significantly in total transcripts, but not in community composition, therefore, gene expression was a direct response to environmental conditions [44]. Comparing membership between guilds previously defined by CAZy expression [44] and guilds defined here by hierarchical clustering of extracellular protease expression revealed guild-dependent trends. In the rhizosphere guilds, comprised primarily of *Gammaproteobacteria* (formerly *Betaproteobacteria*) known to be commonly associated with rhizosphere soil [31, 72, 73], we found high similarity in membership, whereas in the detritusphere guilds there was low similarity. This implies that in the rhizosphere, the same bacteria break down complex carbon and complex nitrogen, whereas in bulk soil these processes are performed by different taxa. The functional guilds identified here exhibited a pattern of temporal succession throughout plant maturation, where expression of protein degrading enzyme genes in the rhizosphere could be influenced by the host plant through control of exudation, or alternatively, may be a response to competition with the plant for labile nitrogen. Moreover, an analysis of a different subset of this experimental data [44], showed that microbial community composition (by 16S rRNA gene sequencing) in each experimental group changed very little compared to gene expression over the course of this experiment [44]. Therefore, the changing expression patterns over time that we observed are likely related to changes in environmental conditions or cues, such as macromolecular N availability and inorganic N availability, as opposed to extensive shifts in community composition.

The identification of key rhizosphere players is the first step toward in situ inoculation. Inoculation of seeds with plant growth promoting bacteria, or bioaugmentation, has shown promise in reducing the need for fertilizers [74] and improving plant biomass [75] as well as rhizosphere available nitrogen [76]. Augmentation of soil or seeds with bacteria that specialize in macromolecular organic N degradation has been suggested as a way to reduce the use of fertilizers [77]. Ideally, bioaugmentation should use bacteria endemic to the specific plant and soil in order to provide strong competitors [78]. The different extracellular protease expression patterns that we observed amongst various taxonomic groups indicate that potential plant-beneficial partners change throughout root growth. Hence, the functional guild characterization approach that we used could be a useful way to decide which taxa should be selected for bioaugmentation, with rhizosphere- and aging root-specific taxa as candidates. Even within a functional guild, there may be bioaugmentation candidates suitable for different conditions. For example, a phylotype of *Gammaproteobacteria* (formerly *Betaproteobacteria*) belonging to the rhizosphere guild had higher extracellular protease expression in the presence of root detritus, whereas a *Burkholderiales* taxon, *Janthinobacterium* sp. and *Massilia* sp. expressed protease genes more highly in unamended rhizosphere. Organisms from different guilds or with different temporal expression patterns, such as *Rhizobacter* and *Janthinobacterium*, could be combined into consortia that should effectively contribute more than a single taxon to the nitrogen economy of the plant through mineralization of nitrogen coinciding with plant demand over time.

## Conclusions

In this study, we revealed lineage-specific spatiotemporal patterns of protease and chitinase gene expression by soil bacteria in the presence of live and dead roots. Using a replicated time-series we demonstrate several key points: (1) chitinase is expressed mostly by eukaryotes near dead roots, likely to predate on fungi feeding on root litter, (2) patterns of expression of protease that are confounded by total gene expression emerge with lineage-specific analysis, particularly in the rhizosphere, (3) these patterns also highlight interactions within the microbiome such as activity of predatory bacteria, (4) bacteria that express protease genes in the rhizosphere also target complex C, whereas in bulk soil complex N is targeted by different bacteria than those who target complex C. Finally, we propose that bacteria that naturally express protease in the rhizosphere may be excellent candidates for bioaugmentation to reduce fertilizer use, particularly if a consortium could be developed that expresses these genes at different stages in the rhizosphere lifespan.

## Supplementary information

Supplementary figures

Supplementary tables

## Data Availability

The R code used in this work is publicly available at https://github.com/ellasiera/Protease_ISME_2022. All data used in this publication, including raw reads and genome collection, is publicly available. Metagenomes assembled MAGs can be found in NCBI PRJNA517182, stable isotope probing MAGs in http://ggkbase.berkeley.edu/, single amplified genomes in IMG under study name Mediterranean Grassland Soil Metagenome and single amplified genomes in IMG, see sup. Table S2 in Nuccio et al., 2020 for accession numbers [44]. Raw reads can be found in JGI IMG. For JGI ID’s (accession numbers), see Supplementary Table S1 in Nuccio et al. 2020 [44].
