# Correction to: Expression of macromolecular organic nitrogen degrading enzymes identifies potential mediators of soil organic N availability to an annual grass

**DOI:** 10.1038/s41396-023-01441-w

**Published:** 2023-05-31

**Authors:** Ella T. Sieradzki, Erin E. Nuccio, Jennifer Pett-Ridge, Mary K. Firestone

**Affiliations:** 1 Department of Environmental Science, Policy and Management, University of California, Berkeley, CA USA; 2 Physical and Life Sciences Directorate, Lawrence Livermore National Laboratory, Livermore, CA USA; 3 Life & Environmental Sciences Department, University of California Merced, Merced, CA USA; 4 Present Address: Laboratoire Ampère, École Centrale de Lyon, Lyon, France

**Keywords:** Microbial ecology, Biogeochemistry

Correction to: *The ISME Journal* 10.1038/s41396-023-01402-3, published online 14 April 2023

The original online version of this article was revised:

The sentence ‘However, this change in expression was not reflected in changes in overall microbial community composition’ in this article, should have read ‘In addition, overall microbial community composition changes at a slower rate compared to functional gene expression.’

The original article has been corrected.

