# Supplementary material for: Expression of macromolecular organic nitrogen degrading enzymes identifies potential mediators of soil organic N availability to an annual grass
Source: ISME J. 2023 Apr 14;17(7):967–75. doi: 10.1038/s41396-023-01402-3 (PMC10284887; doi:10.1038/s41396-023-01402-3)

Supplementary figure S1: Schematic of the experimental design (adapted with permission from Nuccio et al., 2020 26). White boxed represent bulk soil bags made of root-excluding mesh. Dashed black lines aroung the roots denote the rhizosphere.

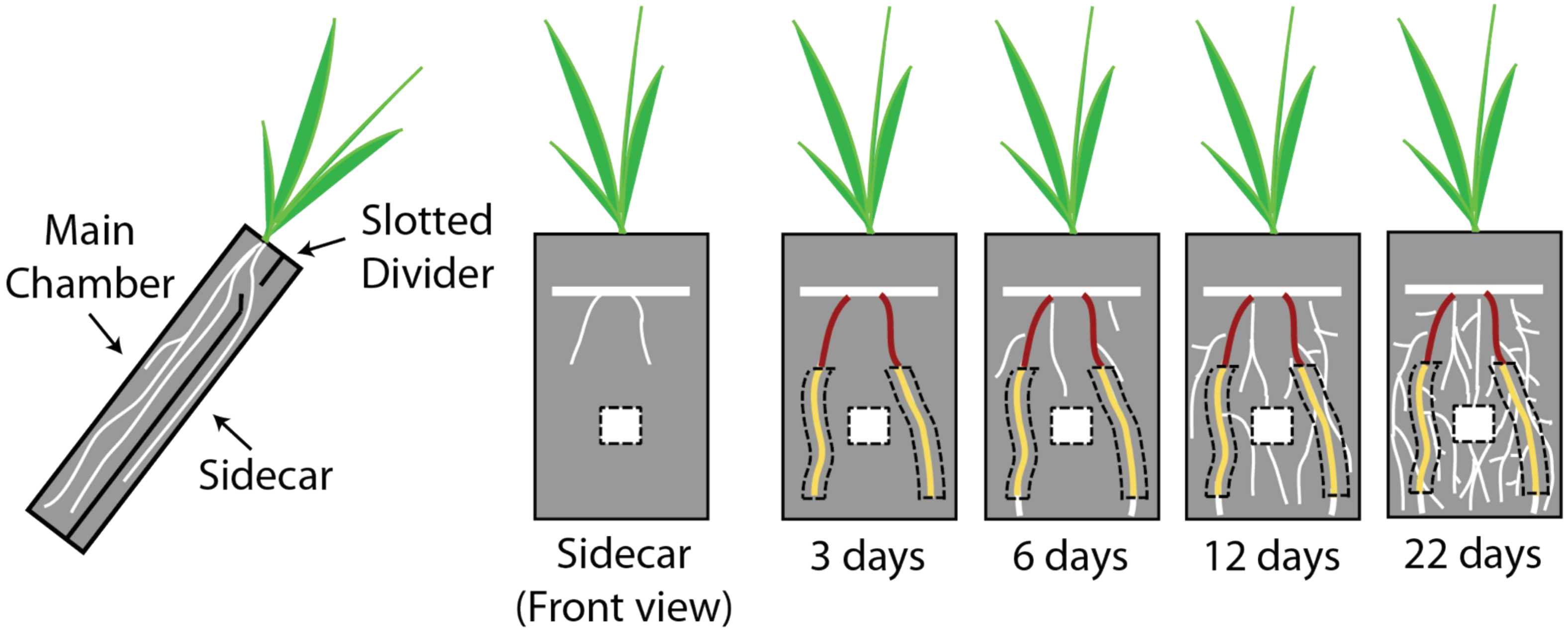

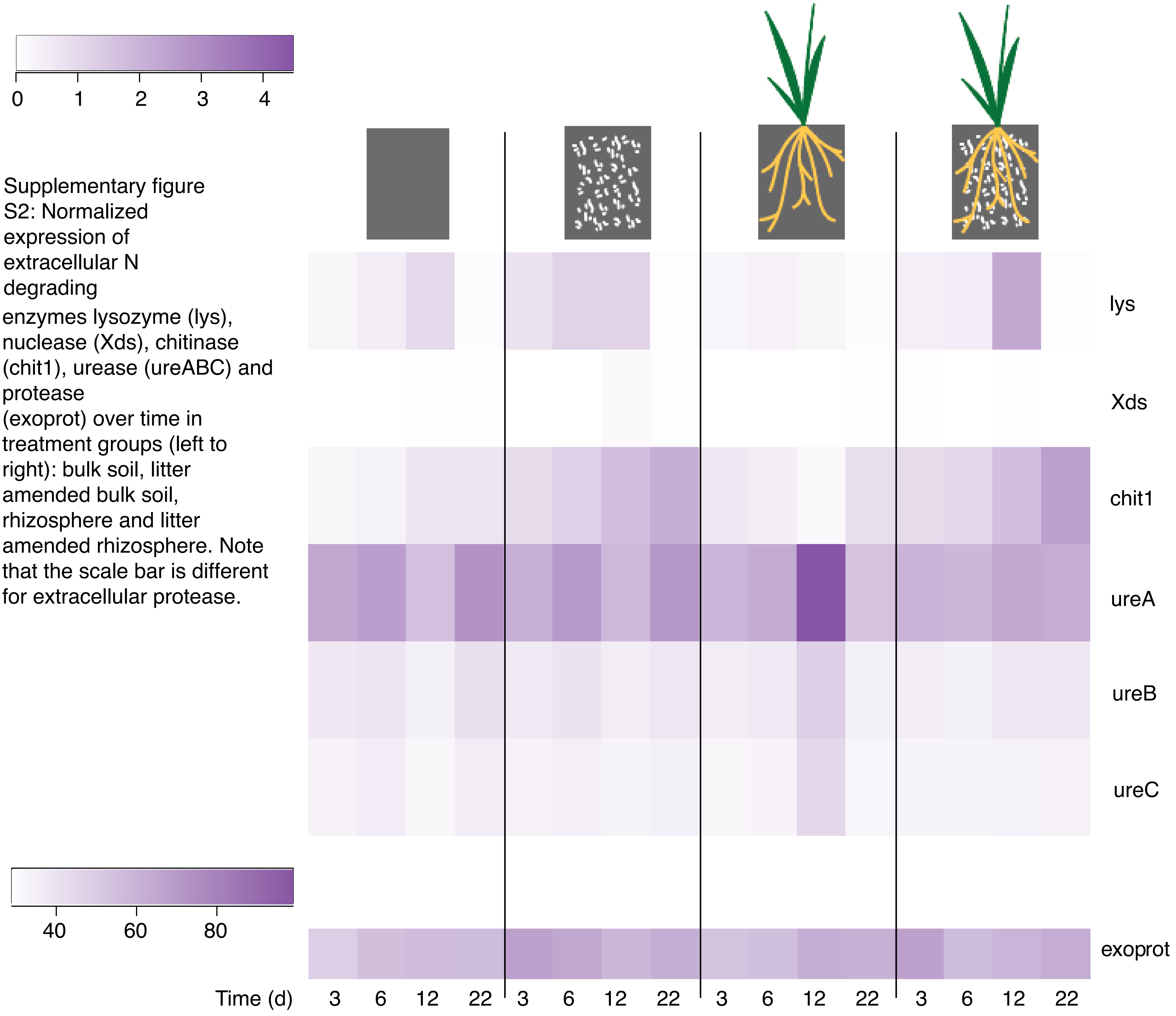

Supplementary figure S2: Normalized expression of extracellular N degrading enzymes lysozyme (lys), nuclease (Xds), chitinase (chit1), urease (ureABC) and protease (exoprot) over time in treatment groups (left to right): bulk soil, litter amended bulk soil, rhizosphere and litter amended rhizosphere. Note that the scale bar is different for extracellular protease.

Supplementary figure S3: Taxonomic disctribution of protease gene expression by phylum, location (rhizosphere or bulk soil), litter amendment and structural protease group

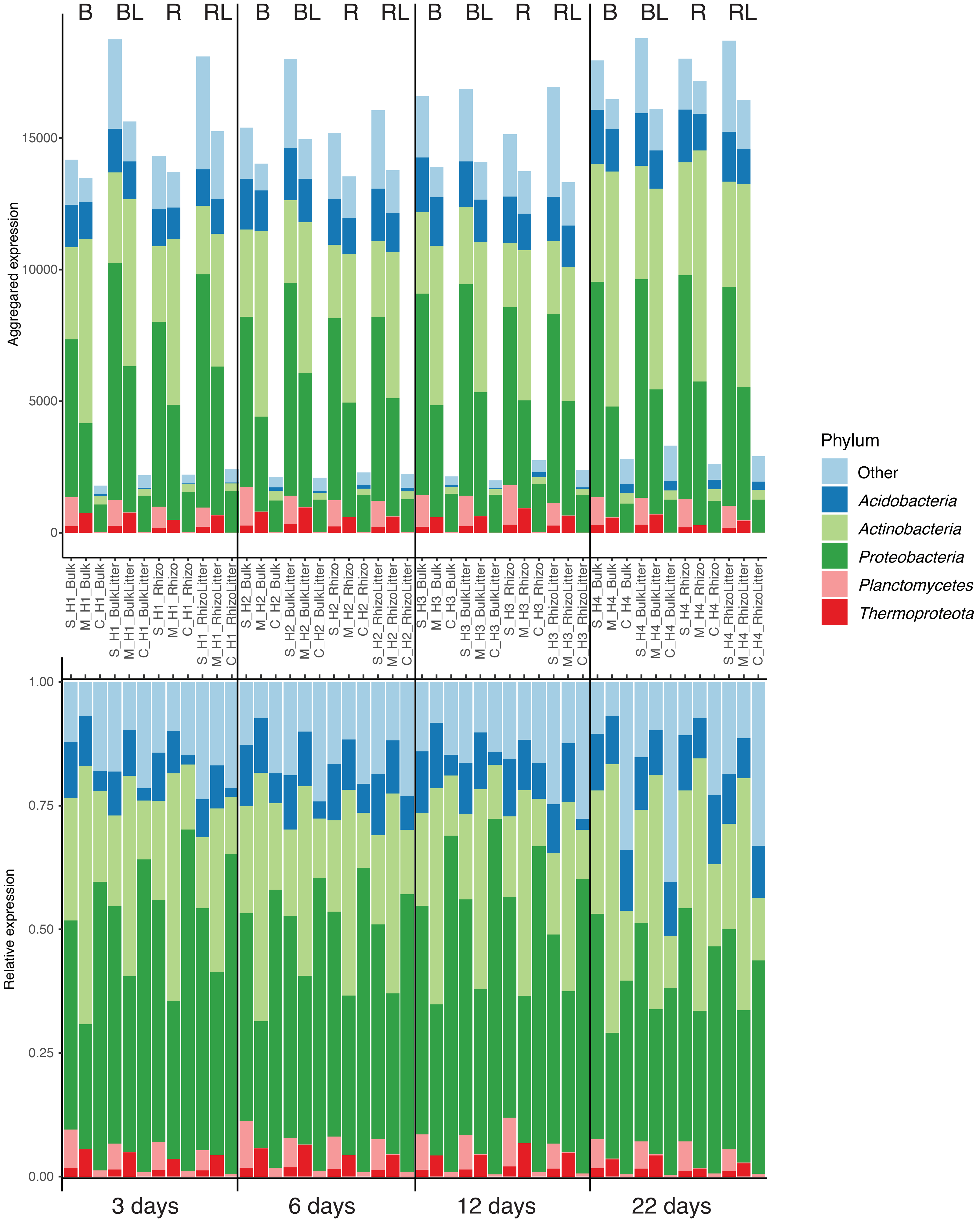

Supplementary figure S4: Aggregated normalized expression of extracellular proteases by phylum (log transformed) with hierarchical clustering.

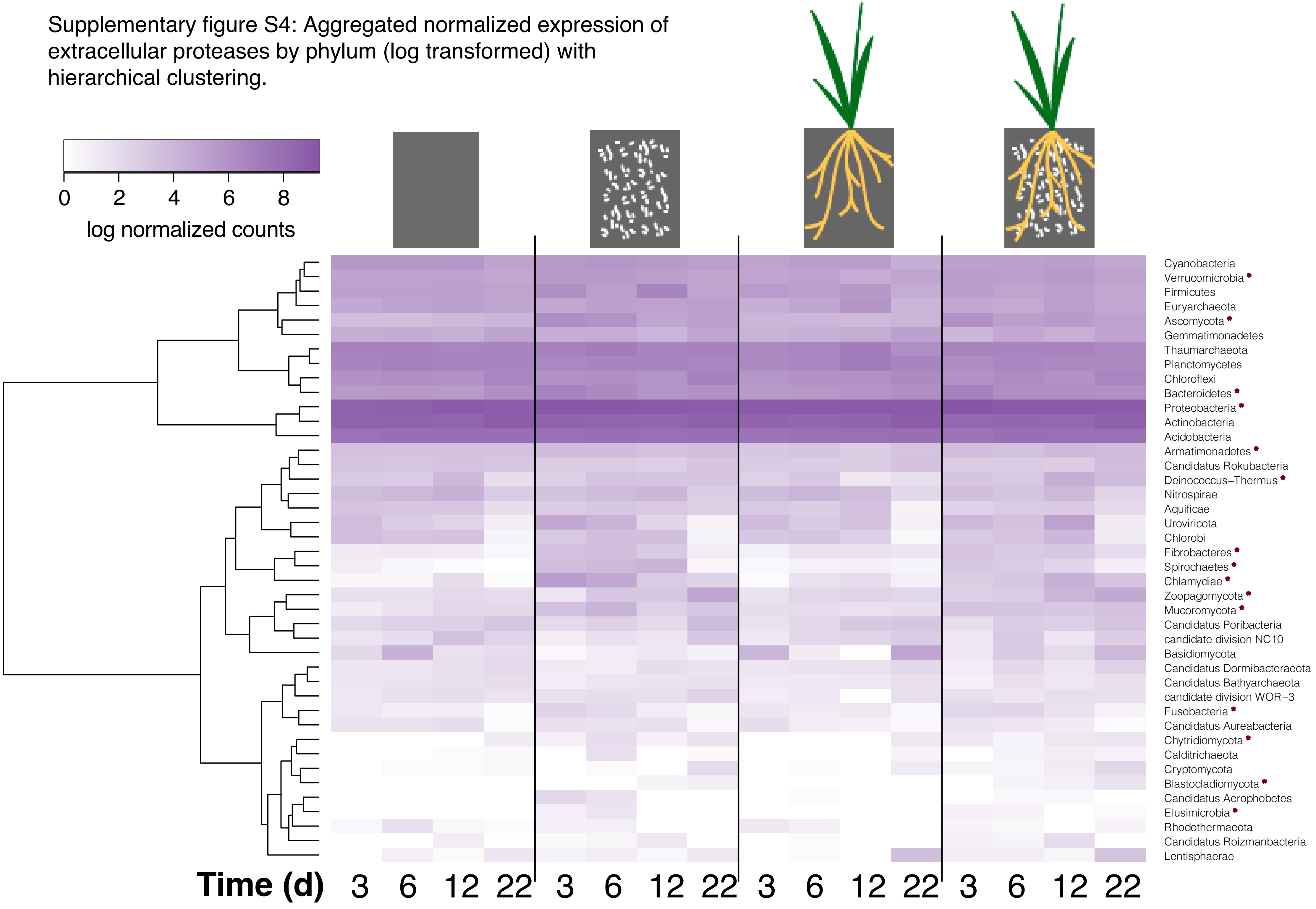

Supplementary figure S5: Aggregated normalized expression of extracellular proteases by class (log transformed) with hierarchical clustering.

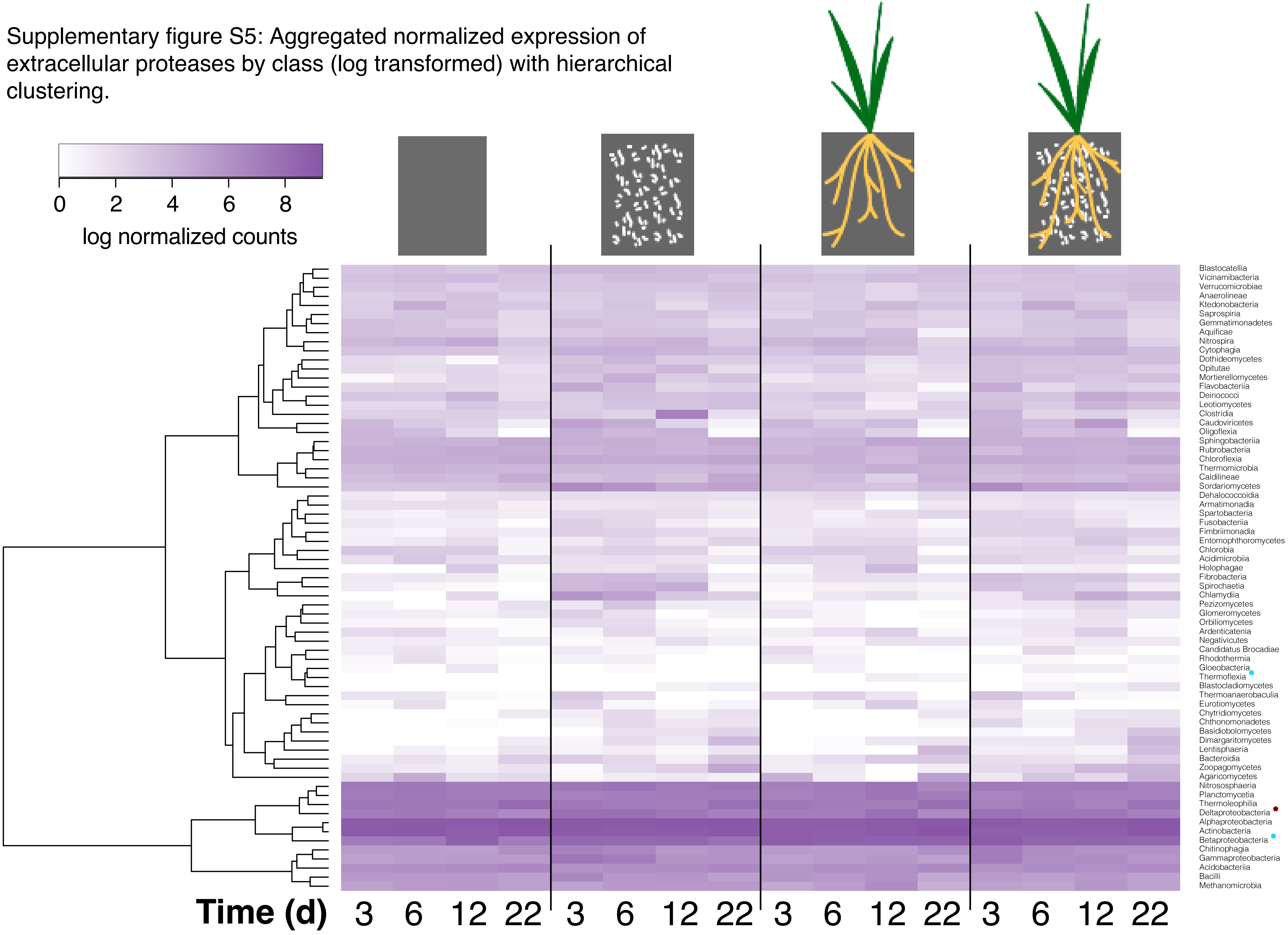

Supplementary figure S6: Aggregated normalized expression of extracellular proteases by order (log transformed) with hierarchical clustering.

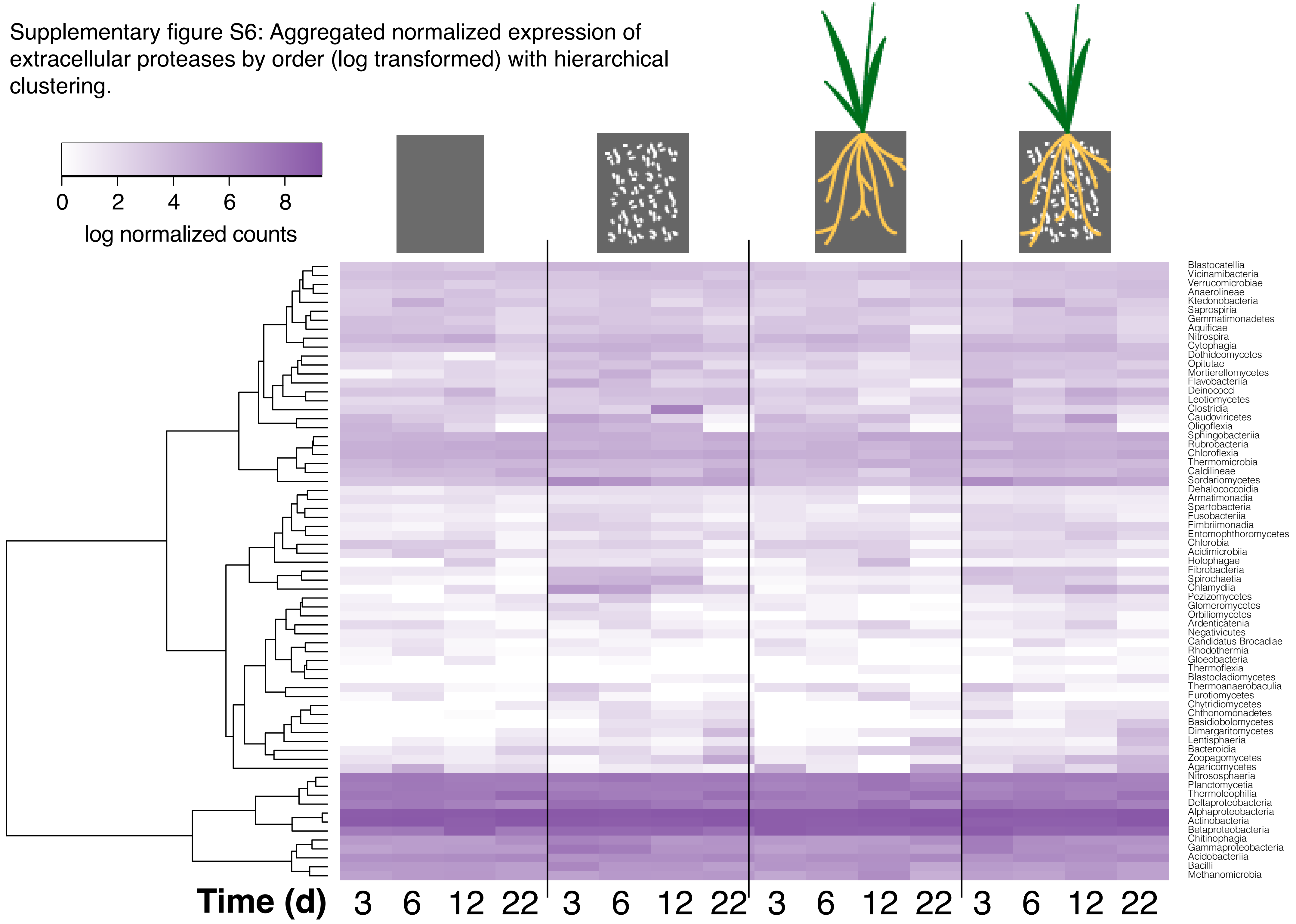

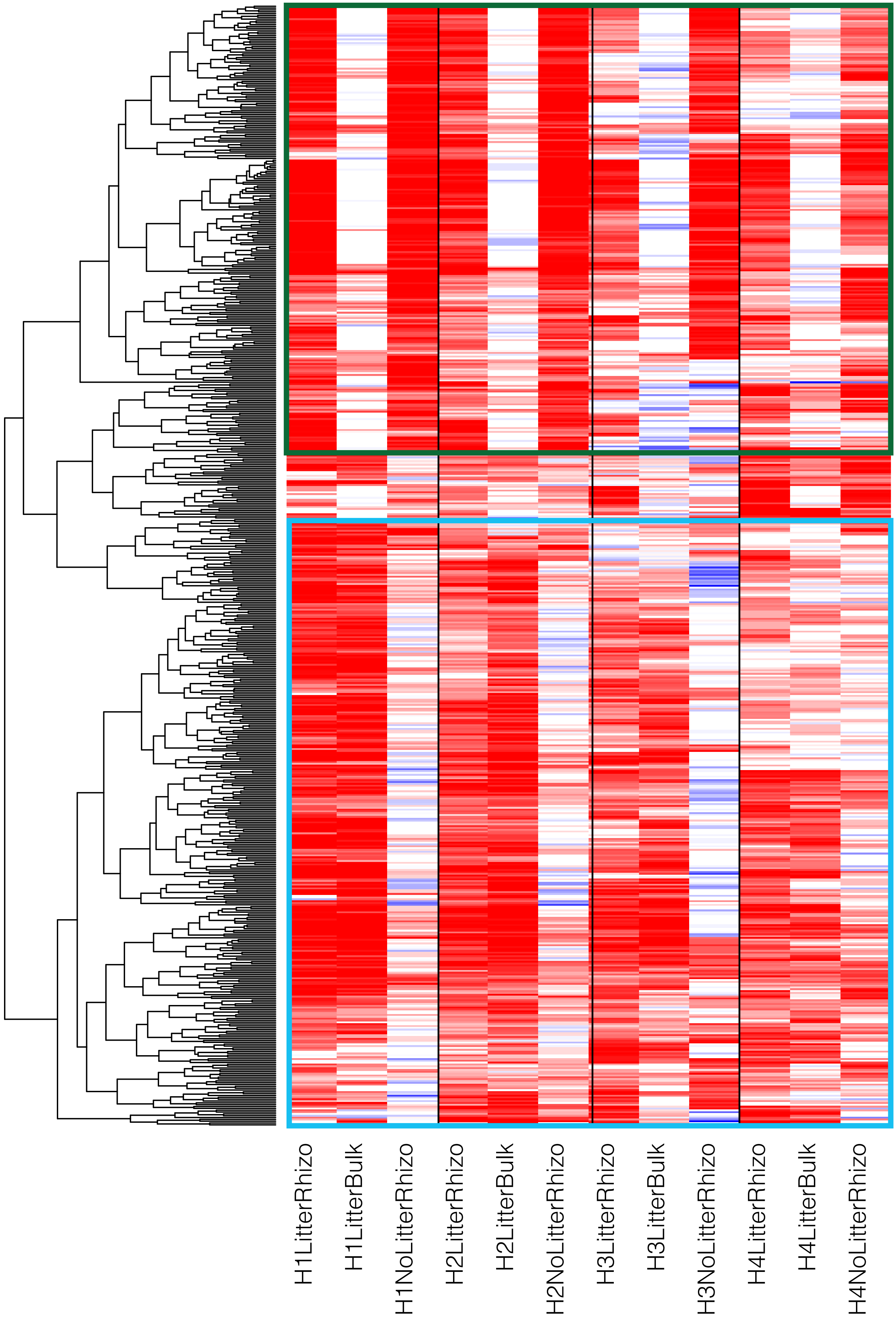

Supplementary figure S7: Functional guilds determined by upregulation of extracellular protease open reading frames assembled from metatranscriptomes. Green = rhizosphere guild, cyan = detritusphere guild

Supplementary figure S8: Number of overlapping genomes between guilds defined by extracellular protease and guilds defined by carbohydrate active enzymes (CAZy).

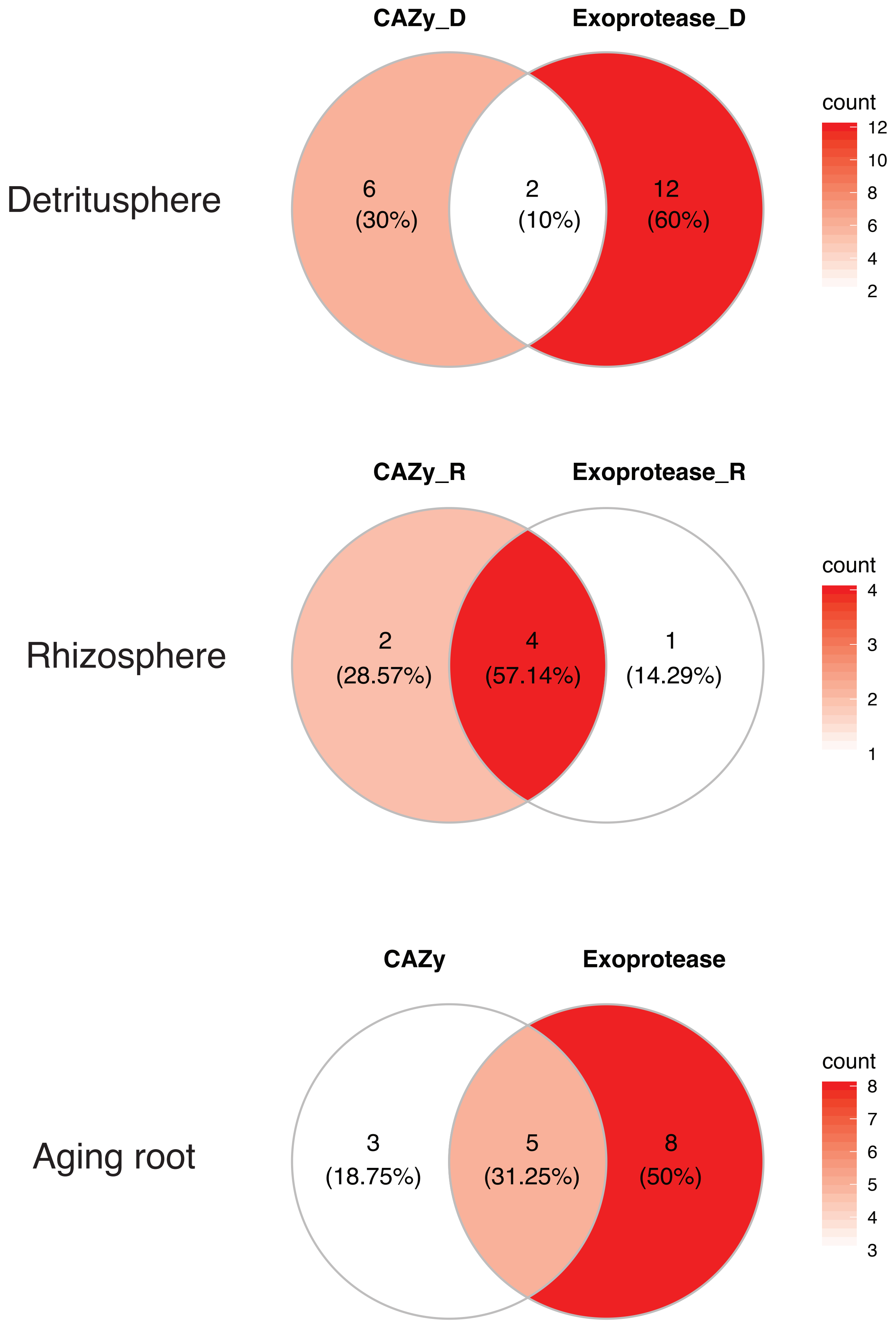

Supplement: Supplementary file 1 — Supplementary figures [file 41396_2023_1402_MOESM1_ESM.pdf]
